# Endoscopist specialty is associated with colonoscopy quality

**DOI:** 10.1186/1471-230X-13-78

**Published:** 2013-05-03

**Authors:** Mengzhu Jiang, Maida J Sewitch, Alan N Barkun, Lawrence Joseph, Robert J Hilsden

**Affiliations:** 1 Division of Clinical Epidemiology, Research Institute of the McGill University Health Centre, 687 Pine Avenue West, V Building, Room V2.15, Montreal, QC, H3A 1A1, Canada; 2 Department of Medicine, McGill University, Montreal, QC, Canada; 3 Division of Gastroenterology, McGill University Health Centre, Montreal, QC, Canada; 4 Department of Epidemiology, Biostatistics and Occupational Health, McGill University, Montreal, QC, Canada; 5 Department of Medicine, University of Calgary, Calgary, AB, Canada; 6 Department of Community Health Sciences, University of Calgary, Calgary, AB, Canada

**Keywords:** Colonoscopy quality, Polypectomy, Adenoma detection rate, Specialty

## Abstract

**Background:**

Some studies have shown that endoscopist specialty is associated with colorectal cancers missed by colonoscopy. We sought to examine the relationship between endoscopist specialty and polypectomy rate, a colonoscopy quality indicator. Polypectomy rate is defined as the proportion of colonoscopies that result in the removal of one or more polyps.

**Methods:**

A cross-sectional study was conducted of endoscopists and their patients from 7 Montreal and 2 Calgary endoscopy clinics. Eligible patients were aged 50–75 and covered by provincial health insurance. A patient questionnaire assessed family history of colorectal cancer, history of large bowel conditions and symptoms, and previous colonoscopy. The outcome, polypectomy status, was obtained from provincial health administrative databases. For each city, Bayesian hierarchical logistic regression was used to estimate the odds ratio for polypectomy comparing surgeons to gastroenterologists. Model covariates included patient age, sex, family history of colorectal cancer, colonoscopy indication, and previous colonoscopy.

**Results:**

In total, 2,113 and 538 colonoscopies were included from Montreal and Calgary, respectively. Colonoscopies were performed by 38 gastroenterologists and 6 surgeons in Montreal, and by 31 gastroenterologists and 5 surgeons in Calgary. The adjusted odds ratios comparing surgeons to gastroenterologists were 0.48 (95% CI: 0.32–0.71) in Montreal and 0.73 (95% CI: 0.43–1.21) in Calgary.

**Conclusions:**

An association between endoscopist specialty and polypectomy was observed in both cities after adjusting for patient-level covariates. Results from Montreal suggest that surgeons are half as likely as gastroenterologists to remove polyps, while those from Calgary were associated with a wide, non-significant Bayesian credible interval. However, residual confounding from patient-level variables is possible, and further investigation is required.

## Background

Colorectal cancer (CRC) screening programs are either in preparation or ongoing in all Canadian provinces and many developed countries [1]. Colonoscopy is central to CRC screening, and colonoscopy quality assurance initiatives are underway in several countries [2-4]. Measures of quality in colonoscopy, such as cecal intubation rate, colonoscope withdrawal time, and adenoma detection rate (ADR) [5,6], vary substantially among endoscopists [7-10]. The establishment of benchmarks for these quality indicators aims to standardize colonoscopy practice quality.

Of the many quality indicators identified, only ADR has been shown to independently predict CRC diagnosis after colonoscopy, an important public health outcome that suggests failure of screening [11]. However, ADR is difficult to assess using population-based data due to the lack of pathology report information in health administrative databases. Polypectomy rate, the proportion of colonoscopies that result in the removal of one or more polyps, has been proposed as an alternative quality indicator because it is known at the time of colonoscopy, strongly correlated with ADR, and available from health administrative data [12-14]. Quality benchmarks for polypectomy rates have been proposed [12,14].

Some studies have shown endoscopist specialty to be associated with cancers missed by colonoscopy [15-18], raising concerns about training and quality assurance. Polypectomy rate is an upstream marker of quality because failure to remove and diagnose pre-cancerous and cancerous lesions leads to missed cancers. It is conceivable, therefore, that differences in missed cancers between specialties may originate with specialty differences in polypectomy rates. The relationship between endoscopist specialty and polypectomy rate has not been examined. If this relationship echoes that between specialty and missed cancers, this finding would lend support to the usefulness of polypectomy rate as a quality indicator.

The objectives of the present study were to determine whether there is a difference in polypectomy rates between surgeons and gastroenterologists beyond what is attributable to differences in patient risk profiles, and to assess the variability in polypectomy rates within each specialty.

## Methods

### Study design

We conducted a cross-sectional analysis combining data from 2 prospective cohort studies. Recruitment for the first cohort occurred between January and March 2007 in Montreal and Calgary for the purpose of developing an administrative data algorithm to identify screening colonoscopies. The second cohort was recruited between January 2008 and March 2009 in Montreal to provide additional subjects for the purpose of the present study. Seven hospitals participated in Montreal: Royal Victoria Hospital, Montreal General Hospital, St. Mary’s Hospital Centre, Jewish General Hospital, Hôpital Maisonneuve-Rosemont, Hôpital Fleury, and Centre hospitalier de l’Université de Montréal. Two institutions participated in Calgary: Foothills Medical Centre and Peter Lougheed Centre. The same data collection methods were used for both cohorts and at all sites. Ethics approval was obtained from the McGill University Faculty of Medicine Institutional Review Board and the research ethics boards at each study site.

### Data collection

Participating physicians were staff endoscopists at the study hospitals with provincial health insurance billing privileges for colonoscopy. A research assistant approached consecutive patients who were waiting for their colonoscopy with a study physician in the endoscopy waiting room on select days. Eligible patients were aged 50–75 and covered by provincial health insurance. Reasons for not being covered by provincial health insurance include being in the military or RCMP, being a treaty status Indian, and being a resident of another province. The reason for this exclusion criterion is that we were only able to link to provincial records in Quebec and Calgary.

The research assistant explained the study, obtained consent, and administered a brief questionnaire to patients on socio-demographics, history of gastrointestinal conditions, large bowel symptoms, previous CRC screening tests, and family history of CRC.

Data on polypectomy status were obtained from physician billing records from the Régie de l’Assurance Maladie du Québec (RAMQ) and Alberta Health and Wellness (AHW). The Alberta ambulatory care database also provided polypectomy status for Albertan patients. For patients who underwent more than one colonoscopy during the study period, we included only the first visit in the analyses.

### Statistical analyses

Bayesian hierarchical logistic regression models were fitted to estimate the association between endoscopist specialty and polypectomy rate. This technique accounts for endoscopist-level clustering, and allowed us to estimate the odds ratio at the endoscopist level, while adjusting for patient-level risk factors for CRC and adenoma, including: age (50-54/55-59/60-64/65-70/70-75), sex, family history of CRC (y/n), previous colonoscopy (y/n), and colonoscopy indication (screening/non-screening). Screening was defined as no history of large bowel symptoms (rectal bleeding, unintentional weight loss, abdominal pain) in the past 6 months, and no history of gastrointestinal conditions (polyp, CRC diagnosis, inflammatory bowel disease, and previous bowel surgery). To estimate the variability of polypectomy rates within each specialty, endoscopist-specific rates were computed from random intercepts for endoscopists. Covariates were centered around their respective means, so that the inverse logits of the intercepts yielded endoscopist-specific rates for typical patients. All analyses were conducted using WinBUGS software version 1.4.3 (MRC Biostatistics Unit, Cambridge). Diffuse or wide prior distributions were used for all parameters in all models. Ninety-five percent credible intervals, the Bayesian equivalents of the frequentist confidence intervals, are indicated by CrI.

We took measures to account for the imperfect accuracy of health administrative data. For Quebec, we adjusted polypectomy rates using previously estimated sensitivity, 84.7% (95% CI: 79–89%), and specificity, 99.0% (95% CI: 98–100%), of the RAMQ polypectomy billing code [19]. The adjustment was done within the WinBUGS model, where the adjusted rates were calculated from the rates estimated from the logistic model [20]. For Alberta, overlapping polypectomy data sources were available from both the AHW billing data and the ambulatory care data. We considered a patient to have had a polypectomy if the polypectomy code appeared in either database; this a conservative approach because administrative codes tend to have good specificity but poor sensitivity [19,21]. Due to differences in the extent of CRC screening, administrative data quality, and adjustment methods, data from each city were analyzed separately rather than combined in a single model.

## Results

### Patient population

In Montreal, 2,134 (81.6%) of the 2,614 patients approached were eligible and consented to participate. A total of 38 gastroenterologists, 6 surgeons, and 1 internist performed 1,906 (89.3%), 207 (9.7%), and 21 (1%) colonoscopies, respectively. The patients seen by the internist were excluded from further analysis, as the sample size was too small to make inferences about this specialty. Hence 44 endoscopists and 2,113 patients from Montreal were included. The average number of patients per endoscopist was 48, ranging from 4 to 154 among gastroenterologists, and 9 to 86 among surgeons.

In Calgary, 541 (88.1%) of the 614 patients approached were eligible and consented to participate. A total of 31 gastroenterologists and 5 colorectal surgeons performed 444 (82.1%) and 94 (17.4%) colonoscopies, respectively. Three (0.6%) patients were excluded from analysis because their colonoscopies were performed by trainees. The final sample for Calgary included 37 endoscopists and 538 patients. The average number of patients per endoscopist was 15, and ranged from 1 to 73 among gastroenterologists, and from 6 to 30 among surgeons. Table 1 presents characteristics of patients from Montreal and Calgary.

**Table 1:** Patient characteristics by city

	**Montreal**	**Calgary**
**Patient Characteristic**	**N (%)**	**N (%)**
Age, mean (sd)	60.1 (7.1)	59.7 (6.9)
Male	1059 (50.1)	248 (46.1)
Family history of CRC^a^	500 (23.6)	134 (24.9)
Colonoscopy in the past 10 years	988 (46.8)	189 (35.1)
Screening^b^	855 (40.5)	208 (38.7)
History of gastrointestinal conditions^c^	583 (27.6)	143 (26.6)
Large bowel symptoms in the past 6 months^d^	840 (39.8)	211 (39.2)
**Total**	**2113**	**538**

^a^ CRC: colorectal cancer.

^b^ Screening was defined as no history of gastrointestinal conditions and no large bowel symptoms in the past 6 months.

^c^ History of gastrointestinal conditions includes polyp, CRC diagnosis, inflammatory bowel disease, and previous bowel surgery.

^d^ Large bowel symptoms include rectal bleeding, unintentional weight loss, abdominal pain in the past 6 months.

### Results of hierarchical logistic regression

The hierarchical logistic regression results with polypectomy as the outcome for Montreal and Calgary are shown in Table 2. The odds ratios for polypectomy with surgeons as compared to gastroenterologists, adjusted for patient age, sex, family history of CRC, indication (screening vs. non-screening), and previous colonoscopy were 0.48 (95% CrI: 0.32–0.71) in Montreal and 0.73 (95% CrI: 0.43–1.21) in Calgary.

**Table 2:** Odds ratio estimates for polypectomy from hierarchical logistic regression models for Montreal and Calgary

	**Montreal**	**Calgary**
**Variable**	**OR**^**a **^**(95% CrI)**	**OR**^**a **^**(95% CrI)**
**Endoscopist Level**		
Surgical specialty^b^	0.48 (0.32–0.71)	0.73 (0.43–1.21)
**Patient Level**		
Age Category		
50–54	ref	ref
55–59	1.58 (1.17–2.15)	1.37 (0.82–2.31)
60–64	1.49 (1.09–2.04)	1.50 (0.84–2.62)
65–69	2.00 (1.45–2.78)	1.85 (0.96–3.356)
70–75	2.14 (1.53–3.01)	2.00 (1.06–3.76)
Male	1.85 (1.51–2.26)	1.93 (1.31–2.81)
Family History of CRC^c^	1.11 (0.87–1.41)	1.32 (0.84–2.07)
Colonoscopy in the past 10 years	0.75 (0.60–0.93)	0.68 (0.44–1.03)
Screening^d^	0.86 (0.69–1.07)	0.79 (0.53–1.18)

^a^ Odds ratio adjusted for all other covariates in the model.

^b^ The reference category is gastroenterology.

^c^ CRC: colorectal cancer.

^d^ Screening was defined as no history of gastrointestinal conditions and no large bowel symptoms in the past 6 months. The reference category is non-screening, which was defined as having a history of gastrointestinal conditions or large bowel symptoms.

**Figure 1:**
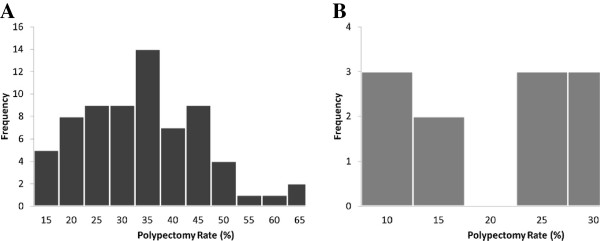
**Histograms of endoscopist-specific polypectomy rates estimated from hierarchical logistic regression models among A) gastroenterologists and B) surgeons.** Models were adjusted for patient age (50-54/55-59/60-64/65-70/70-75), sex, family history of colorectal cancer, colonoscopy in the past 10 years, and colonoscopy indication (screening was defined as no history of gastrointestinal conditions and no lower abdominal symptoms in the past 6 months.). Covariates were centered such that the rates are interpretable as endoscopist-specific rates for typical patients.

To illustrate variability of polypectomy rates, endoscopist-specific polypectomy rate estimates from hierarchical logistic regression for each specialty are shown in Figure 1. The estimates ranged from 6.0% (95% CrI: 0.30–19.6%) to 28.6% (95% CrI: 15.6–46.2%) among surgeons and from 12.3% (95% CrI: 3.9–25.9%) to 62.1% (95% CrI: 45.6–78.9%) among gastroenterologists.

## Discussion

Using a combination of primary and health administrative data from Montreal and Calgary, we estimated the association between endoscopist specialty and polypectomy rate. We found a clinically important difference between endoscopist specialities in Montreal, with surgeons being approximately 50% less likely than gastroenterologists to remove polyps. A similar trend was observed in Calgary but wide credible intervals preclude a definitive conclusion. Patient risk factors yielded odds ratios consistent with the literature, including increase in the probability of polypectomy with increasing age. However, despite statistical adjustment, residual confounding due to patient-level risk factors may contribute the observed difference between specialties.

Several prior studies have examined the association between endoscopist speciality and missed CRCs. In a retrospective cohort study using population level data from Ontario, Bressler et al. identified endoscopist specialty as a risk factor for incident CRCs post-colonoscopy among both male and female patients [16]. In contrast to our study, the difference was found mainly between gastroenterologists and primary care physicians, rather than between gastroenterologists and surgeons. This study also found that office colonoscopies are more likely to be associated with missed lesions than hospital-based colonoscopies. A Manitoba study also showed no difference in missed cancers between surgeons and gastroenterologists. This model included colonoscopy volume and practice location (rural vs. urban) as covariates, neither of which was significantly associated with missed cancers [18]. Rabeneck et al. reported an odds ratio of 1.39 (95% CI: 1.16–1.67) for the risk of CRC diagnosis after negative colonoscopy in the patients seen by surgeons vs. those seen by gastroenterologists in the hospital setting [17]. Colonoscopy volume was not a predictor of missed cancers in this study. Baxter et al. used administrative data from Ontario and found no difference between surgeons and gastroenterologists in post-colonoscopy CRCs, but indicated that non-surgeon and non-gastroenterologist specialties were at increased risk for missed cancers [15]. Similar to previous findings, practice setting was associated with post-colonoscopy CRCs, while colonoscopy volume was not. Only one of the aforementioned studies that used post-colonoscopy CRCs as an outcome detected a difference between surgeons and gastroenterologists. Although many of these studies indicated a non-significant trend towards higher rates of missed cancers among surgeons, the failure to detect a statistically significant difference may have been due to a lack of power because the outcome is rare. In contrast to province-wide studies from Ontario and Manitoba on endoscopy specialty and quality, our study was restricted to urban hospitals and to ambulatory care patients; thus, our findings are less generalizable. Nevertheless, in province-wide studies, the power to detect an interaction between location and specialty may be of concern, since the majority of colonoscopies are performed by gastroenterologists in urban areas and by surgeons in rural areas [22].

We determined the variability in polypectomy rates within each specialty, and found considerable variation in both specialties. Several studies have reported important variation in ADRs by specialty. Barclay et al. examined 2,053 screening colonoscopies by 12 endoscopists and found that ADRs varied from 9.4 to 32.7% [23]. Chen et al. studied the variation among 9 endoscopists who performed 10,034 colonoscopies. After adjusting for patient age and sex, detection rates for at least one adenoma ranged between 15.5 and 41.1% [24]. Imperiale et al. found that ADRs ranged from 7% to 44% among 46 endoscopists who performed 2,664 screening colonoscopies [7]. Our results mirror those of others showing variation in endoscopist performance, and further current knowledge in that considerable variation exists between as well as within specialties.

Our results suggest a clinically important difference in polypectomy rates between surgeons and gastroenterologists after adjusting for patient-level factors, as well as substantial variation among all endoscopists. Some of this variation may be explained by endoscopist-level factors such as training, practice factors, and technical factors. Recently, performance quality was compared in trainees with similar endoscopy experience; gastroenterology trainees outperformed surgery trainees on several quality indicators including colonoscopy completion rate, polypectomy rate, ADR, and withdrawal time [10]. One Calgary study found that gastroenterology trainees performed considerably more colonoscopies than surgery trainees during their training [25]. Further, all of the gastroenterology fellows fulfilled the minimum number of colonoscopies recommended by the American Society for Gastrointestinal Endoscopy for assessment of competency, while none of the surgery residents did. These findings suggest that differences in colonoscopy quality between the two specialties may arise from differences in training.

Practice factors such as setting and annual case volume may also contribute to variation among endoscopists. For example, Bressler et al. and Baxter et al. both found non-hospital based colonoscopies to be associated with increased risk of missed cancers [15,16]. A recent study from the U.K. showed that volume and accreditation were significantly associated with colonoscopy quality indicators [26]. Interestingly, in this study surgeons had a higher polyp detection rate compared to physicians. However, this difference was minimal after adjustment for patient-level factors. Studies in Manitoba and Ontario have not found colonoscopy volume to predict missed cancers [15,17,18].

In terms of technical factors, withdrawal time has been identified as a significant predictor of ADR in some studies. Results from the Bowel Cancer Screening Programme in England show a significant increase in ADR among endoscopist with mean withdrawal time of 11 minutes or longer compared to those with mean withdrawal time of less than 7 minutes [27]. Investigators in Spain examined bowel cleansing, sedation, cecal intubation, and withdrawal time as potential predictors of ADR. Only withdrawal time longer than 8 minutes was independently associated with ADR [28]. However, a German study found neither annual case volume nor withdrawal time to be correlated with ADR [29]. The mixed findings regarding practice and technical factors that affect colonoscopy quality may be due to differences in training, accreditation, practice settings, and CRC screening delivery models between countries.

A major strength of our study is primary data collection on patient level CRC risk factors that enabled adjustment for colonoscopy indication, family history of CRC and previous colonoscopy. This is important because polypectomy rate is a function of both patient risk and endoscopist performance.

One study limitation is residual confounding. Although we adjusted for many important patient risk factors, it is possible that differences in the characteristics of patients referred to surgeons and gastroenterologists were not adequately captured by the patient-level covariates. A second limitation is potential misclassification, as data on polypectomy status were derived from provincial health administrative databases. To address this issue, we employed methods to adjust for the imperfect accuracy of health administrative data in both provinces. Using health administrative data whilst acknowledging and accounting for its limitations is good practice in clinical and health services research. However, our approach to misclassification adjustment for the Montreal data may have introduced bias if misclassification was differential between surgeons and gastroenterologists. Differences in administrative data quality between specialties may arise from differences in billing practices. Nevertheless, it is reassuring that the Calgary sample, where misclassification was reduced by combining two data sources, showed results in the same direction as the Montreal sample. Thirdly, hierarchical modeling was used to estimate the rates as it has the advantage of conservatively bringing unstable estimates closer to the overall mean so that they are less likely to affect the range of variation in polypectomy rates [30]. While use of this statistical technique allowed us to estimate the polypectomy rates of endoscopists with few study patients, there were wide credible interval limits around some individual estimates.

Although our findings highlight the important issue of the discrepancy in quality between surgeons and gastroenterologists, the lack of endoscopist-level variables in our study preclude us from isolating the modifiable predictors of endoscopist performance. Future studies aimed at teasing out such factors would help inform changes to training, accreditation, and quality assurance programs. We hope that our findings will serve as impetus for such investigations.

## Conclusions

In conclusion, we found that surgeons were less likely to remove polyps compared to gastroenterologists, and that considerable variation in polypectomy rates exists within each specialty. Using polypectomy rate as an indicator of colonoscopy quality, our findings showed a difference in practice quality between the two specialties despite controlling for variations in patient risk profiles. Potential reasons for this discrepancy, such as training, practice volume, and technical factors, need to be investigated in future studies. Our findings suggest that the difference in ADRs may begin at the level of polyp removal, and lend further support for the use of polypectomy rate as a colonoscopy quality indicator.

## Competing interests

The authors declare that they have no competing interests.

## Authors’ contributions

MJ conducted the data analysis and drafted the manuscript. MJS conceived of the study, participated in its design, and helped draft the manuscript. ANB participated in the study design and contributed to the data collection. LJ participated in the study design and advised the analysis. RJH contributed to study data collection. All authors contributed to the interpretation of the findings, and read and approved the final manuscript.

## Pre-publication history

The pre-publication history for this paper can be accessed here:

http://www.biomedcentral.com/1471-230X/13/78/prepub
